# Metabolic Bulk Volume is an independent prognostic factor and facilitates identifying high risk cases for DLBCL patients treated with the R-CHOP

**DOI:** 10.3389/fonc.2026.1747186

**Published:** 2026-02-03

**Authors:** Silu Cui, Panpan Luan, Yuxiao Hu, Qi Jiang

**Affiliations:** 1 Jiangsu Province Hospital of Chinese Medicine, Affiliated Hospital of Nanjing University of Chinese Medicine, Nanjing, Jiangsu, China; 2 Department of PET/CT Center, Jiangsu Cancer Hospital and Jiangsu Institute of Cancer Research and The Affiliated Cancer Hospital of Nanjing Medical University, Nanjing, China

**Keywords:** 18F-FDG PET/CT, diffuse large B-cell lymphoma, metabolic bulk volume, prognosis, total metabolic tumour volume

## Abstract

**Objective:**

The purpose of this study was to evaluate the prognostic value of metabolic bulk volume (MBV), a baseline 18-fluorode-oxyglucose positron emission computed tomography (^18^F-FDG PET/CT) derived indicator characterizing bulky disease, in DLBCL patients treated with R-CHOP.

**Methods:**

311 consecutive newly diagnosed Diffuse Large B-Cell Lymphoma (DLBCL) patients were retrospectively evaluated. Estimating MBV, Distance between the centers of the two farthest lesions (Dmax) and Total metabolic tumour volume (TMTV) as semiquantitative metabolic parameters. Receiver Operating Characteristic (ROC) curve analysis was used to determine the optimal cut-off values. Progression-Free Survival (PFS) and Overall Survival (OS) were the endpoints for evaluating prognosis. PFS and OS were estimated using Kaplan-Meier curves, and comparisons were performed via log-rank test.

**Results:**

Multivariate analysis showed that only two baseline ^18^F-FDG PET factors, MBV and Dmax, remained significant for OS (P = 0.004 and P < 0.0001). Combining high MBV and high Dmax generated three risk groups for PFS (P < 0.0001) and OS (P < 0.0001). This was equally effective as the three-risk-group stratification by high TMTV combined with high Dmax for both PFS (P < 0.0001) and OS (P < 0.0001). Further stratification of the three risk groups generated by the combination of high TMTV and high Dmax using MBV showed that MBV could further stratify PFS in both the low-risk group (P < 0.0001) and high-risk group (P = 0.015), as well as OS in high-risk group (P = 0.001).

**Conclusion:**

MBV was an independent prognostic factor for DLBCL patients. The combination of MBV with parameters reflecting tumor dissemination distribution or total tumor burden further improved the risk stratification for staging in DLBCL patients.

## 1 Introduction

Diffuse large B-cell lymphoma (DLBCL) represents the most common histological subtype among aggressive lymphomas, accounting for approximately 25% of all non-Hodgkin lymphomas (1). The standard first-line therapy, comprising rituximab, cyclophosphamide, doxorubicin, vincristine, and prednisone (termed the R-CHOP regimen), is effective in 60%-70% of patients (2). Although international guidelines have explicitly incorporated polatuzumab-based regimens into the first-line treatment recommendations for DLBCL to address the limitations of the R-CHOP regime (3, 4), approximately one-third of patients cannot be cured by standard immunochemotherapy regimens and progress to relapsed or refractory disease (2, 5). Patients with relapsed or refractory disease typically have a significantly reduced life expectancy, as the response rate of salvage therapy regimens is extremely low (5). Thus, early risk stratification remains necessary. Current prognostic scoring systems, primarily the International Prognostic Index (IPI) (6) and the National Comprehensive Cancer Network IPI (NCCN-IPI) (7), fail to accurately identify high-risk patients (7–9).

Over the past five years, the prognostic role of quantitative ^18^F-FDG PET/CT parameters in baseline DLBCL patients, particularly total metabolic tumor volume (TMTV) (10–13) and the index characterizing the dissemination of the two farthest lesions (Dmax) (14–16), has been validated in DLBCL cohorts. TMTV, the cumulative volume of all ^18^F-FDG-avid tumor regions, more accurately reflects tumor burden than clinical factors (e.g., Ann Arbor staging and/or LDH levels) and provides a more comprehensive assessment of tumor burden (17). However, TMTV does not account for the spatial distribution of lesions throughout the body. Dmax, defined as the distance between the two farthest lesions, represents the spatial dissemination of the tumor. Combining TMTV with Dmax has been shown to improve the prognostic predictive value in DLBCL patients (15). Patients with high tumor burden and distant spatial tumor dissemination have a higher risk of treatment failure and shorter survival compared to those with low tumor burden and close spatial tumor dissemination. However, these two factors do not account for the role of bulky disease in the prognosis of DLBCL patients. Bulky single tumor has been demonstrated to be a poor prognostic factor in various malignancies, including lung cancer (18), nasopharyngeal carcinoma (19), prostate cancer (20), and thyroid cancer (21). Similarly, bulky single disease has been shown to be an adverse prognostic predictor in DLBCL patients (22–24) and has been recommended for clinical use as an additional prognostic indicator for lymphoma patients alongside the IPI and NCCN-IPI (23, 25). Patients with bulky disease have a worse prognosis, possibly due to poorer absorption of chemotherapeutic agents in bulky lesions (23).

Previous studies have commonly used the maximum tumor diameter (MTD), defined as the longest diameter of the largest lesion, for imaging-based measurement of bulky disease (22–25). However, earlier research has demonstrated that rituximab-based therapy diminishes the prognostic value of MTD in DLBCL (24). Moreover, recent studies have shown that when using 18-fluorode-oxyglucose positron emission computed tomography (^18^F-FDG PET/CT) to measure the metabolic bulk volume (MBV) of the largest lesion to characterize bulky disease, the presence of high MBV enhances the prognostic value of TMTV in DLBCL patients (26). Additionally, MBV serves as a valuable volumetric prognostic indicator for stage II/III DLBCL patients treated with R-CHOP (27), whereas MTD does not. This discrepancy may arise because MTD, a unidimensional measurement, fails to fully represent bulky disease. In contrast, MBV, by measuring the entire volume of bulky lesions, provides more comprehensive information about tumor burden than the maximum diameter, making it better suited for characterizing bulky disease in the era of R-CHOP therapy. However, in the era of R-CHOP as first-line therapy for DLBCL patients, the relationship between MBV and clinical indices such as the IPI, NCCN-IPI scores, as well as baseline ^18^F-FDG PET/CT parameters characterizing other tumor features (e.g., Dmax and TMTV), remains unclear.

The purpose of this study was to analyze MBV as a novel baseline ^18^F-FDG PET/CT marker for characterizing bulky disease, determine its predictive value in DLBCL patients treated with R-CHOP, explore its relationship with the IPI, NCCN-IPI scores and their components in prognostic prediction, and to test its incremental predictive value over Dmax and TMTV.

## 2 Materials and methods

### 2.1 Study populations

**Figure 1:**
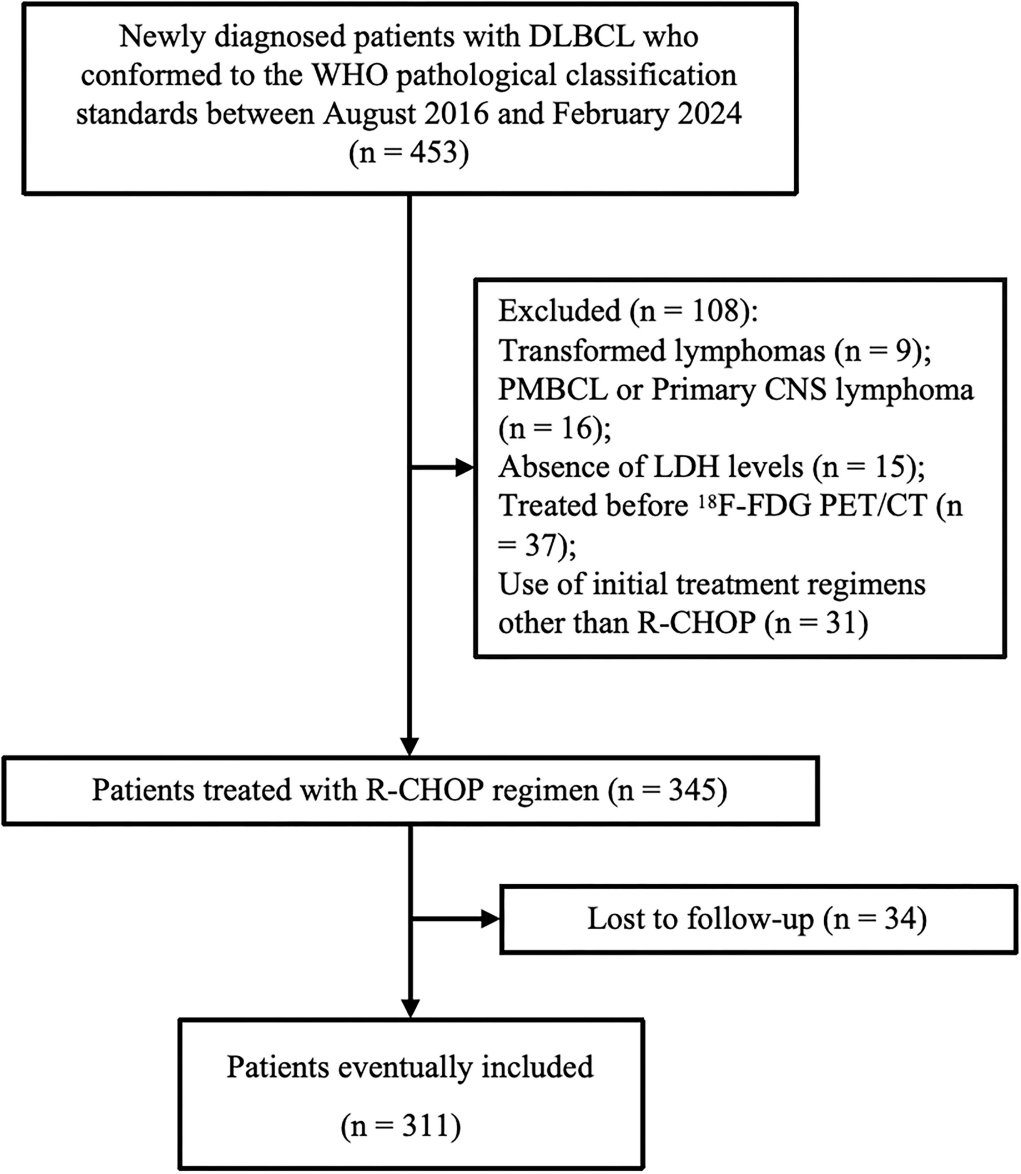
Flow chart of patient selection (PMBCL, primary mediastinal B-cell lymphoma; CNS, central nervous system).

This retrospective, single-center study included consecutive newly diagnosed DLBCL patients who underwent baseline ^18^F-FDG PET/CT scans at Jiangsu Cancer Hospital between August 2016 and February 2024. Subsequently, all patients received first-line standard R-CHOP or R-CHOP-like regimens in accordance with the NCCN Guidelines (28), adhering to the predefined inclusion criteria. **Figure 1** shows the flow diagram of inclusion and exclusion criteria for this study. The study was conducted in accordance with the Declaration of Helsinki (as revised in 2013). This study was approved by the Ethics Committee of Jiangsu Cancer Hospital. Given that this was a retrospective study, informed consent was not required.

Baseline clinical characteristics, including age, serum LDH levels, Eastern Cooperative Oncology Group performance status (ECOG PS), Ann Arbor stage, and extranodal involvement (EN), were collected by reviewing patients’ electronic medical records for calculation of the IPI and NCCN-IPI scores. Based on above scoring system, patients were further categorized into low-risk (NCCN-IPI < 4) and high-risk (NCCN-IPI ≥ 4) groups, as published by Zhou et al. (7) in 2014.

### 2.2 ^18^F-FDG PET/CT image acquisition

Anonymized baseline PET image data in DICOM format were collected for functional parameter measurement. Nuclear medicine physicians analyzed the PET data using LIFEx software (version 5.10, http://www.lifexsoft.org) (29). Lesion localization and size were recorded. TMTV was defined as the sum of metabolic volumes of individual lesions, calculated based on supervised segmentation of tumor regions. According to the recommendations of the European Association of Nuclear Medicine (EANM) guidelines (30), 41% SUVmax threshold was used to delineate regions of interest (ROIs). MBV was defined as the metabolic tumor volume of the largest lesion, similarly delineating ROIs using 41% SUVmax threshold. MTD was defined as the maximum long diameter of the largest lesion. Additionally, the highest SUVmax and mean SUVmean across all lesions were recorded. Finally, the location of each lesion center was used as the lesion’s position, and the Euclidean distance between lesion centers was calculated to determine the distance between the two farthest lesions, defined as Dmax.

### 2.3 ^18^F-FDG PET/CT image analysis

All patients underwent imaging with an integrated PET/CT system (Discovery 710, GE Medical Systems, Waukesha, Wisconsin, USA). After fasting for ≥ 6 h, patients received intravenous ^18^F-FDG at 0.1–0.2 mCi/kg. Blood glucose was measured pre-injection to ensure < 11 mmol/L. During radiotracer distribution, patients rested in a quiet, comfortable room, with oral hydration of 1,000ml water, and emptied their bladder immediately before scanning. Combined imaging was initiated 50–70min post-18F-FDG injection. CT was acquired from vertex to mid-thigh with parameters: 140 kV, Auto mA (noise index 28.5), 0.8 s rotation time, 3.75mm slice thickness.

A PET scan was performed with identical parameters: emission scan time 2 min/bed position, scanning range 6–7 bed positions. PET images were iteratively reconstructed via the ordered-subsets expectation maximization algorithm (CT-based attenuation correction) with the following settings: sharp IR + VUE Point FX (fully 3D iterative reconstruction), 192×192 matrix, 24 subsets/2 iterations, 6.4 post-filter. Transaxial, sagittal, coronal, and fused images were analyzed on GE Healthcare’s Advanced Workstation AW 4.6 (New Jersey, USA).

### 2.4 Follow-up

All included patients were followed up during their visits or the electronic medical records, with the follow-up cutoff date being April 30, 2025, or patient death. The primary endpoints were overall survival (OS) and progression-free survival (PFS). OS was defined as the interval from pathological diagnosis to death from any cause or the last follow-up. PFS was defined as the interval from pathological diagnosis to tumor progression or death from any cause or the last follow-up.

### 2.5 Statistical analysis

Statistical analyses were performed using SPSS Statistics software (Version 23.0). Continuous variables with normal distribution were presented as mean ± standard deviation (SD), while skewed distributions were expressed as median (interquartile range, Q1-Q3). Categorical variables were described by frequency or percentage (%). Spearman’s rank correlation coefficient (ρ) or Pearson’s correlation coefficient (r) was used to test the relationships between ordinal or binary IPI variables, respectively. Optimal cutoffs for semiquantitative ^18^F-FDG PET/CT metabolic parameters were determined by receiver operating characteristic (ROC) curve analysis for PFS and OS. Kaplan-Meier survival analysis was used to estimate PFS and OS, with log-rank tests applied to compare survival differences between groups. Cox regression models were employed to assess the prognostic relevance of baseline metabolic parameters in conjunction with clinical indicators.

## 3 Results

### 3.1 Patient outcome

A total of 311 patients were finally included, with a median follow-up time of 3.2 years (Q1-Q3: 1.8–4.9 years). All patients received R-CHOP or R-CHOP-like regimens as first-line chemotherapy. Progression/relapse occurred in 82 patients at a median time of 11 months (Q1-Q3: 5.75–25.5 months), and 67 patients died at a median time of 17 months (Q1-Q3: 8.0–33 months). The 5-year PFS and OS rates for the entire cohort were 70.4% and 73.6%, respectively. The clinical and imaging characteristics of the patients are shown in **Table 1**. Among all patients, the measured MBV values were greater than the median (13.78) in 155 cases and greater than the mean (68.50) in 49 cases.

**Table 1:** Characteristics of the 311 enroll.

Characteristics	Value
Gender, n (%)	
Male	148 (47.6)
Female	163 (52.4)
Age (years), n (%)	
≤60	147 (47.3)
>60	164 (52.7)
Ann Arbor stage, n (%)	
Stage I/II	153 (49.2)
Stage III/IV	158 (50.8)
LDH, n (%)	
Normal (≤ 245)	213 (68.5)
High (> 245)	98 (31.5)
*EN, n (%)	
0–1	238 (76.5)
> 1	73 (23.5)
ECOG PS, n (%)	
0–1	256 (82.3)
> 1	55 (17.7)
IPl score	
0-2	234 (75.2)
≥3	77 (24.8)
NCCN-IPI	
Low intermediate (0–3)	215 (69.1)
Intermediate high and high (≥4)	96 (30.9)
TMTV (cm3)	
Mean (SD)	116.01 (256.42)
Median (range)	33.03 (0.74–3048.17)
Dmax (cm)	
Mean (SD)	29.20 (24.88)
Median (range)	21.83 (0.01–83.83)
SUVmax (g/ml)	
Mean (SD)	17.37 (11.03)
Median (range)	17.01 (1.38–47.18)
SUVmean (g/ml)	
Mean (SD)	7.87 (6.06)
Median (range)	6.51 (0.07–32.82)
MBV (cm3)	
Mean (SD)	68.50 (208.62)
Median (range)	13.78 (0.83–2648.81)
MTD (cm)	
Mean (SD)	5.07 (3.57)
Median (range)	4.30 (0.5–25.6)

EN, extranodal involvement; LDH, lactate dehydrogenase; ECOG PS, Eastern Cooperative Oncology Group performance status; IPI, International Prognostic Index; NCCN-IPI, National Comprehensive Cancer Network-International Prognostic Index; MBV, metabolic bulk volume; MTD, maximum tumor diameter; TMTV, total metabolic tumour volume; SD, standard deviation.

*Involvement of major organs (bone marrow, central nervous system, liver, gastrointestinal tract, or lung).

### 3.2 Correlations between MBV and other metabolic parameters, Dmax, IPI, NCCN-IPI and components of the IPI

Pearson correlation analysis showed that MBV was very strongly and strongly positively correlated with TMTV and MTD, with ρ of 0.939 and 0.707, respectively (all P < 0.0001). Based on the above observations, we further calculated the variance inflation factor (VIF) to assess collinearity among the aforementioned variables. The results showed no multicollinearity among MBV, TMTV, and MTD, with VIFs of 8.539, 9.236, and 2.174, respectively. MBV showed moderate correlations with NCCN-IPI and LDH. Weak or very weak correlations were observed between MBV and SUVmax, SUVmean, Dmax, IPI, as well as its components (age, EN, Ann Arbor stage, and ECOG PS), as presented in **Supplementary Table 1**. Metabolic parameters measured by ^18^F-FDG PET/CT demonstrated high interobserver agreement (ICC: 0.997, P < 0.001).

### 3.3 ^18^F-FDG PET/CT Features

**Table 2** shows the descriptive statistics of ^18^F-FDG PET/CT characteristics, and **Table 3** presents the results of ROC analysis for each ^18^F-FDG PET/CT parameter.

**Table 2:** ROC analysis of ^18^F-FDG PET/CT features, AUC, sensitivity, and specificity.

Parameter	PFS					OS				
	AUC (95% CI)	Cut-off	Se	Sp	Pvalue	AUC (95% CI)	Cut-off	Se	Sp	Pvalue
TMTV	0.887 (0.845-0.928)	69.54	79.3	84.7	<0.0001	0.869 (0.824-0.914)	69.54	77.6	80.3	<0.0001
Dmax	0.864 (0.821-0.907)	27.56	89	72.5	<0.0001	0.882 (0.843-0.922)	31.23	92.5	73.4	<0.0001
SUVmax	0.59 (0.525-0.656)	14.78	73.2	49.8	0.015	0.599 (0.53-0.669)	14.78	73.1	48.4	0.013
SUVmean	0.607 (0.541-0.673)	4.13	79.3	42.8	0.004	0.599 (0.53-0.668)	4.60	77.6	43.9	0.013
MBV	0.854 (0.807-0.9)	19.36	80.2	75.5	<0.0001	0.842 (0.792-0.892)	48.42	62.1	90.2	<0.0001
MTD	0.803 (0.746-0.859)	4.95	74.4	74.7	<0.0001	0.8 (0.745-0.9)	5.15	73.1	75.4	<0.0001

Se, sensitivity; Sp, specificity. P < 0.05 was considered statistically significant.

**Table 3:** Univariate Cox regression analysis for PFS and OS.

Variable	PFS			OS		
	HR	95% CI	P value	HR	95% CI	P value
Age	1.482	0.951-2.31	0.082	1.872	1.13-3.1	0.015
Ann Arbor Stage	5.44	3.102-9.539	< 0.0001	7.738	3.829-15.634	< 0.0001
LDH	4.686	2.986-7.345	< 0.0001	4.768	2.891-7.864	< 0.0001
EN	4.238	2.746-6.541	< 0.0001	5.228	3.222-8.483	< 0.0001
ECOG PS	2.324	1.465-3.685	0.0003	2.878	1.753-4.725	< 0.0001
IPl score	5.345	3.445-8.295	< 0.0001	7.758	4.677-12.869	< 0.0001
NCCN-IPI	4.954	3.148-7.796	< 0.0001	7.562	4.403-12.988	< 0.0001
TMTV	12.746	7.437-21.846	< 0.0001	10.196	5.725-18.16	< 0.0001
Dmax	14.186	7.083-28.411	< 0.0001	24.036	9.647-59.885	< 0.0001
SUVmax	2.427	1.489-3.959	0.0004	2.37	1.38-4.069	0.002
SUVmean	2.619	1.535-4.47	0.0004	2.622	1.475-4.661	0.001
MBV	8.869	5.129-15.337	< 0.0001	8.911	5.431-14.62	< 0.0001
MTD	6.703	4.068-11.044	< 0.0001	6.967	4.045-11.999	< 0.0001

P < 0.05 was considered statistically significant.

**Figure 2:**
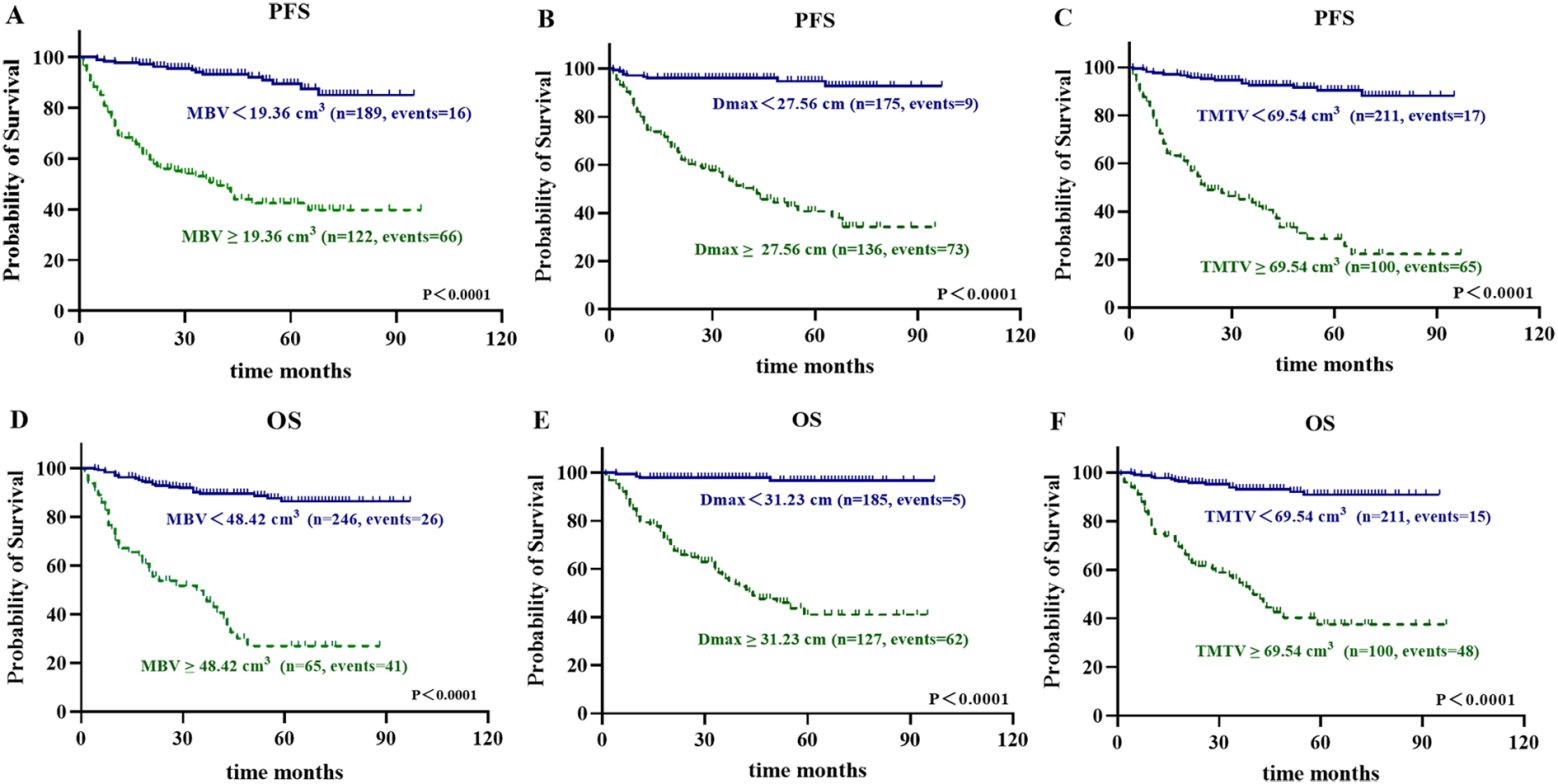
Kaplan–Meier curves depict PFS and OS stratified by MBV **(A, D)**, TMTV **(B, E)**, and Dmax **(C, F)** using cohort-specific cutoff values. Cutoffs for MBV are 19.36 cm³ **(A)** and 48.42 cm³ **(D)**; for TMTV: 27.56 cm³ **(B)** and 31.23 cm³ **(E)**; for Dmax: 69.54 cm **(C, F)**.

Univariate analysis showed that all ^18^F-FDG PET/CT parameters including MBV had predictive value for both PFS and OS. Using the optimal ROC cutoffs, MBV demonstrated a high prognostic capability (P < 0.0001 for PFS and P < 0.0001 for OS) (**Figure 2**), with ROC AUC of 0.853 and 0.840 for PFS and OS, respectively (**Table 2**; **Supplementary Figure 1**). Patients with high MBV had significantly worse prognosis, with 5-year PFS and OS rates of 41.7% and 26.6%, respectively, compared to 89.5% and 86.4% in those with low MBV. For Dmax and TMTV, the ROC AUC for both PFS and OS were consistently > 0.8 (**Table 2**; **Supplementary Figure 1**). Similarly, both Dmax and TMTV demonstrated high prognostic capability (all P < 0.0001 for PFS and OS) (**Figure 2**). Patients with high Dmax and high TMTV had 5-year PFS rates of 40.1% and 28.1%, and 5-year OS rates of 40.7% and 55.6%, respectively. In contrast, those with low Dmax and low TMTV had 5-year PFS rates of 94.7% and 94.6%, and 5-year OS rates of 96.6% and 95.1%, respectively.

Patients with low MBV (< 19.36 cm3 for PFS and < 48.42 cm3 for OS) and high MBV (≥ 19.36 cm3 for PFS and ≥ 48.42 cm3 for OS) showed significant differences in Dmax and TMTV (all P < 0.0001).

### 3.4 Combinations of MBV and Dmax, as well as TMTV and Dmax

In the multivariate Cox regression analysis including MBV, TMTV, and Dmax (**Table 4**), MBV (P = 0.017; hazard ratio [HR]: 2.672), Dmax (P < 0.0001; HR: 6.844), and TMTV (P = 0.003; HR: 3.349) were all significantly associated with PFS. For OS, MBV (P = 0.004; HR: 3.152) and Dmax (P < 0.0001; HR: 11.854) were significantly associated with OS, while TMTV was not (P = 0.638; HR: 1.253).

**Table 4:** Multivariate Cox regression analysis for PFS and OS.

Variable	PFS			OS		
	HR	95% CI	P value	HR	95% CI	P value
Model 1: Individual factors						
Age	–	–	–	1.116	0.646-1.928	0.695
Ann Arbor Stage	1.284	0.672-2.451	0.45	2.197	1.014-4.761	**0.046**
LDH	1.256	0.727-2.17	0.413	1.556	0.864-2.805	0.141
EN	1.598	0.965-2.647	0.069	1.605	0.907-2.841	0.104
ECOG PS	1.142	0.687-1.897	0.609	1.265	0.732-2.187	0.399
TMTV	3.349	1.516-7.396	**0.003**	1.253	0.489-3.213	0.638
Dmax	6.844	3.266-14.341	**< 0.0001**	11.854	4.551-30.877	**< 0.0001**
SUVmax	0.856	0.44-1.667	0.647	0.72	0.337-1.538	0.396
SUVmean	1.209	0.591-2.472	0.604	1.595	0.726-3.507	0.245
MBV	2.672	1.194-5.983	**0.017**	3.152	1.442-6.893	**0.004**
MTD	0.842	0.392-1.812	0.661	1.075	0.407-2.839	0.884
Model 2: MBV and IPI						
MBV	6.283	3.546-11.133	**< 0.0001**	5.284	3.111-8.973	**< 0.0001**
IPl score	2.968	1.876-4.695	**< 0.0001**	4.522	2.632-7.77	**< 0.0001**
Model 3: MBV and NCCN-IPI						
MBV	6.496	3.691-11.434	**< 0.0001**	5.961	3.567-9.96	**< 0.0001**
NCCN-IPI	2.986	1.87-4.767	**< 0.0001**	5.032	2.878-8.796	**< 0.0001**

The values in bold are statistically significant (P < 0.05).

**Figure 3:**
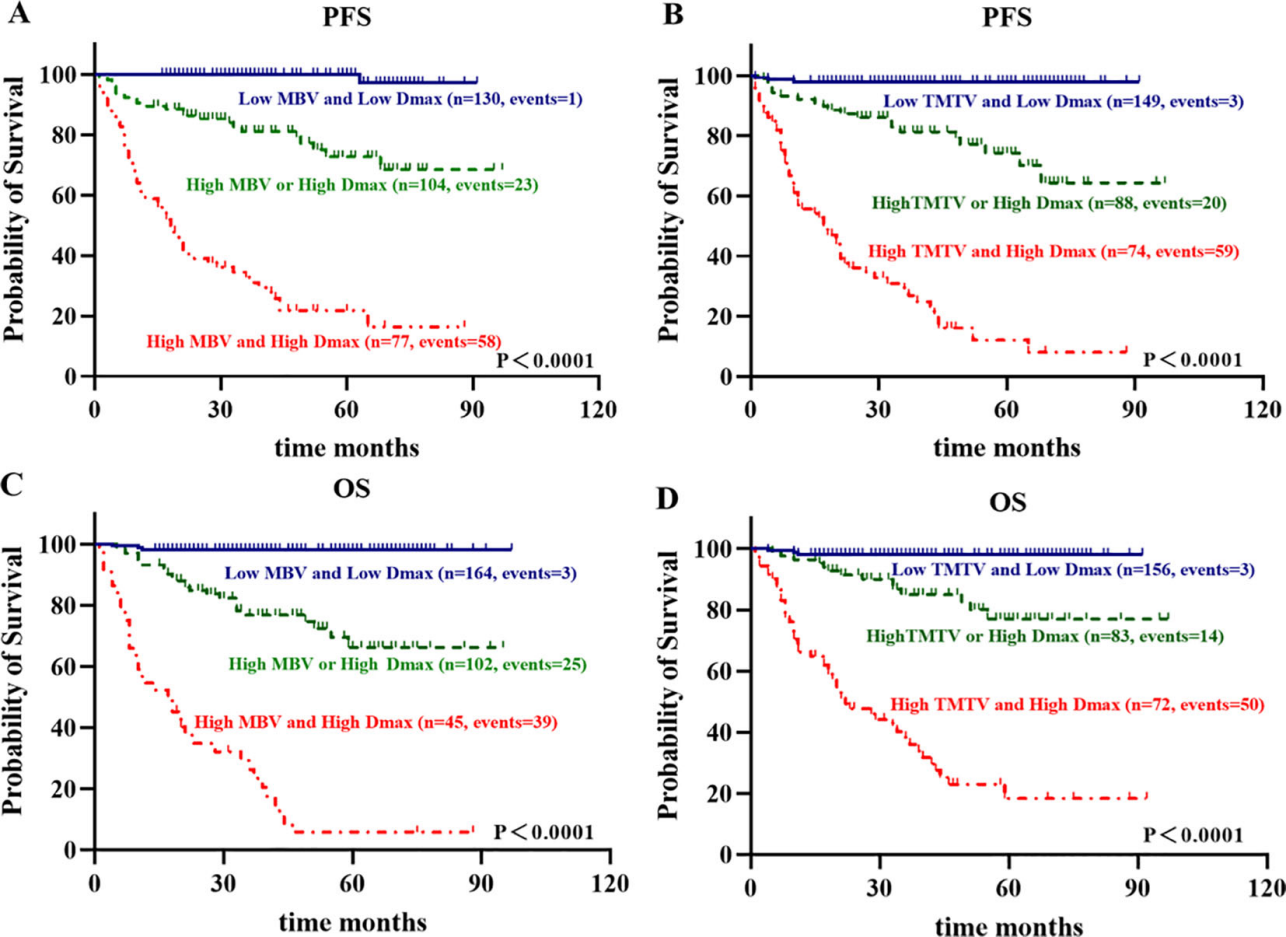
Kaplan–Meier estimates of PFS and OS for baseline MBV-Dmax **(A, C)** Combinations and Baseline TMTV-Dmax **(B, D)** Combinations.

Thus, based on the presence of high MBV (≥ 19.36 cm³ for PFS and ≥ 48.42 cm³ for OS) or high Dmax (≥ 27.56 cm for PFS and ≥ 31.23 cm for OS) risk factors, three risk categories were significantly distinguished (all P < 0.0001 for PFS and OS) (**Figure 3**): Group 1 had no risk factors (n = 130 for PFS and n = 164 for OS), Group 2 had one risk factor (n = 104 for PFS and n = 102 for OS), and Group 3 had both risk factors (n = 77 for PFS and n = 45 for OS). The 5-year PFS rates for the three groups were 100%, 72.7%, and 21.2%, respectively, while the 5-year OS rates were 98.2%, 66.2%, and 5.7%, respectively. Significant differences in both PFS and OS were observed between Group 1 and Group 2, Group 1 and Group 3, as well as Group 2 and Group 3 (all P < 0.0001 for PFS and OS across the three groups).

Similarly, based on the presence of high TMTV (≥69.54 cm³ for both PFS and OS) or high Dmax (≥ 27.56 cm for PFS and ≥ 31.23 cm for OS), three risk categories were significantly distinguished (all P < 0.0001 for PFS and OS) (**Figure 3**): Group 1 had no risk factors (n = 149 for PFS and n = 156 for OS), Group 2 had one risk factor (n = 88 for PFS and n = 83 for OS), and Group 3 had both risk factors (n = 74 for PFS and n = 72 for OS). The 5-year PFS rates for the three groups were 98%, 74.2%, and 28.2%, respectively, while the 5-year OS rates were 98.1%, 76.9%, and 18.1%, respectively. Pairwise comparisons revealed significant differences in both PFS and OS across all three groups: between Group 1 and Group 2, Group 1 and Group 3, and Group 2 and Group 3 (all P < 0.0001 for PFS and OS).

Notably, although combinations of high MBV or high TMTV with high Dmax both showed significant differences in PFS and OS, the combination of MBV and Dmax identified more patients with PFS events (n = 81) in the risk-free subgroup compared to the combination of TMTV and Dmax.

### 3.5 The combination of MBV with the TMTV-Dmax combination

**Figure 4:**
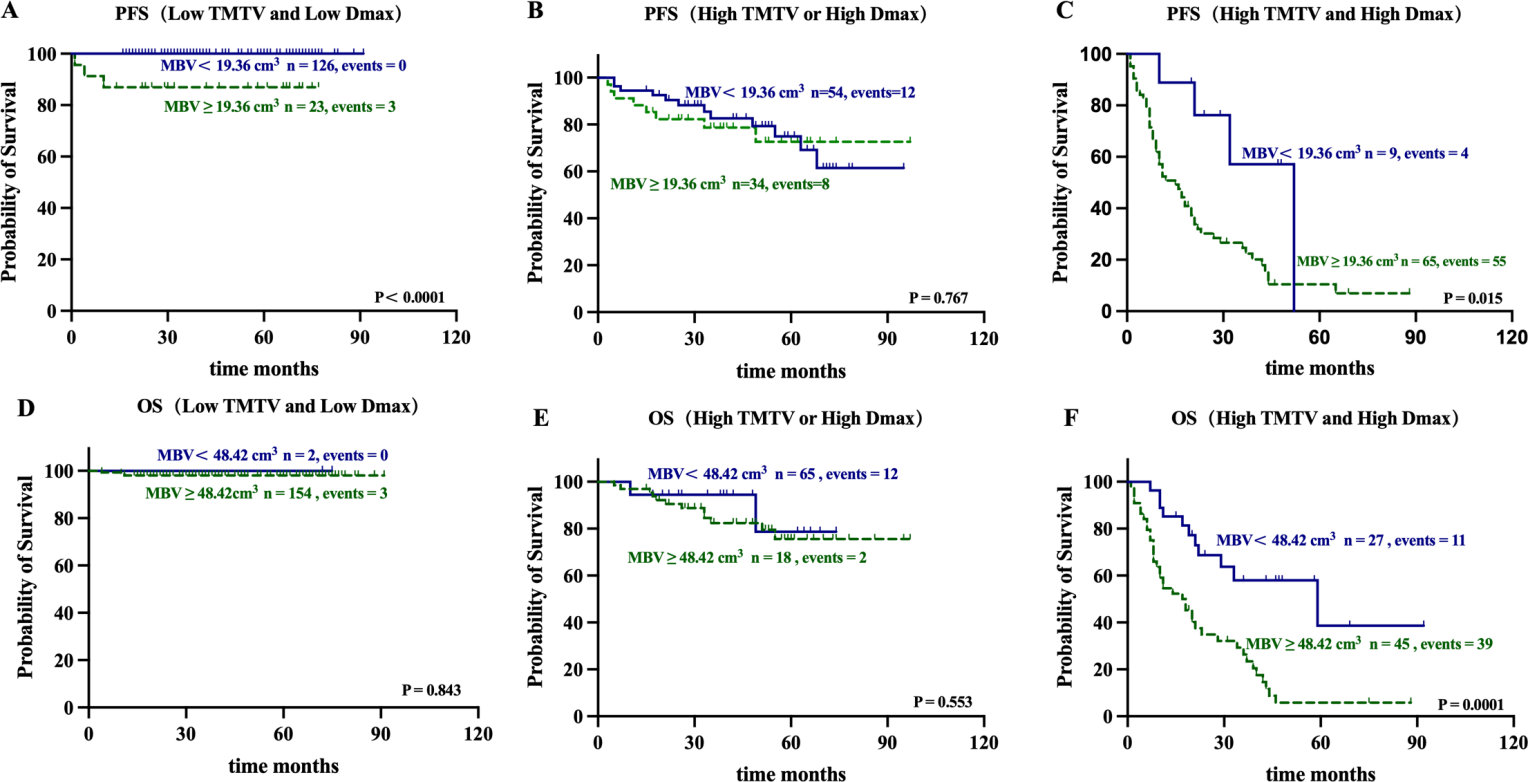
Kaplan–Meier estimates of PFS and OS for MBV (< 19.36cm^3^ vs. ≥ 19.36 cm^3^ and < 48.42 cm^3^ vs. ≥ 48.42 cm^3^, respectively) in TMTV-Dmax Combination Stratification (low TMTV and low Dmax: **(A, D)**; high TMTV or high Dmax: **(B, E)**; high TMTV and high Dmax: **(C, F)**.

**Figure 5:**
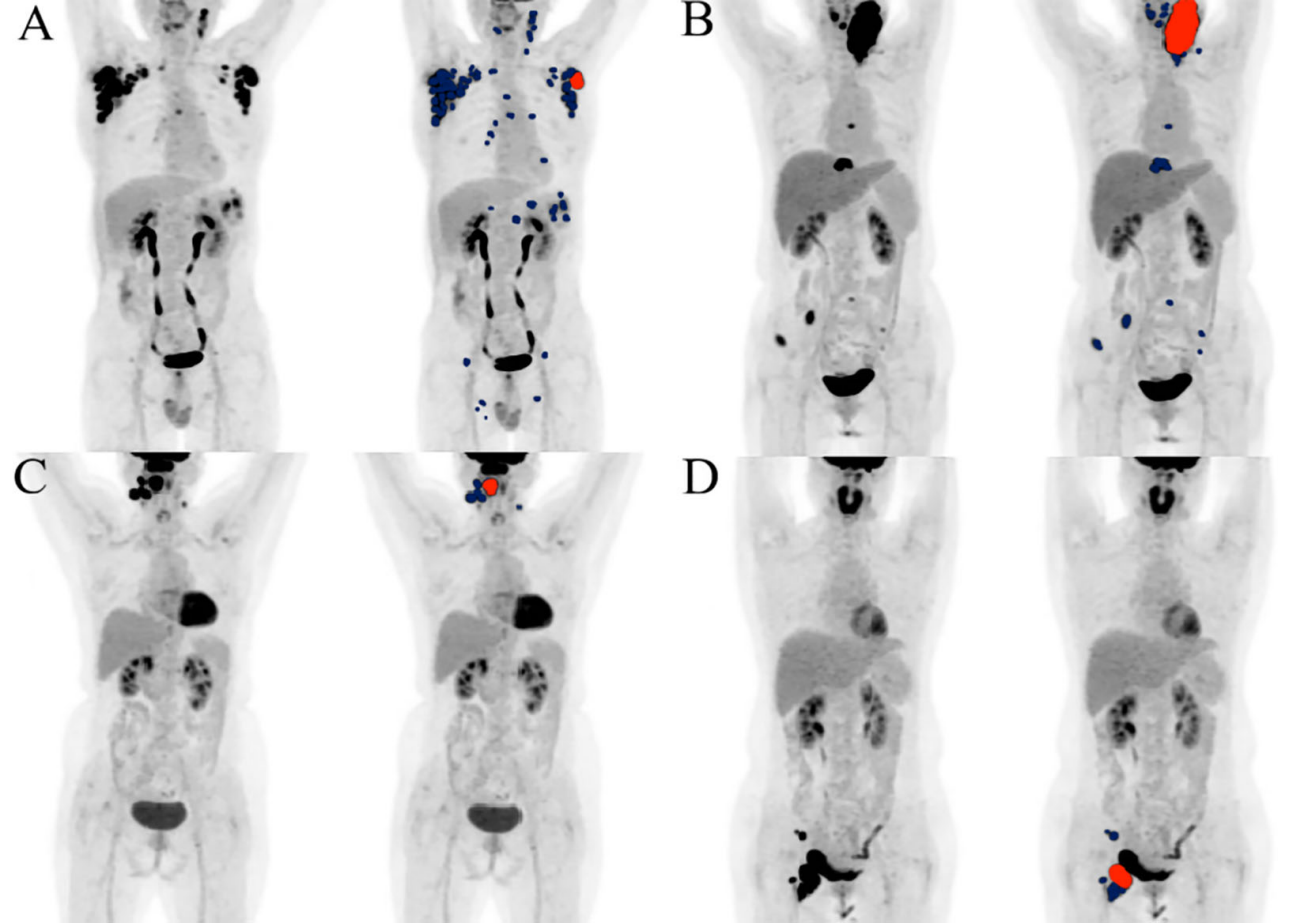
TMTV and Dmax from ^18^F-FDG PET/CT assess tumor burden and dissemination, respectively. MBV reclassified risk in two representative patients **(A, B)** with both high TMTV and high Dmax, as well as in another two **(C, D)** with both low TMTV and low Dmax. All involved lesions are indicated in blue, and the largest lesion used to derive MBV is highlighted in red. Patient **(A)** had high TMTV (99.71 cm³), high Dmax (68.8 cm), and low MBV (11.74 cm²), remaining progression-free and alive after 29 months of follow-up. Patient **(B)** showed high TMTV (100.23 cm³), high Dmax (59.24 cm), and high MBV (82.89 cm²); progression occurred at 3 months and death at 7 months of follow-up. Patient **(C)** had low TMTV (21.04 cm³), low Dmax (9.73 cm), and low MBV (6.30 cm²), remaining progression-free and alive after 83 months of follow-up. Patient **(D)** showed low TMTV (33.03 cm³), low Dmax (10.20 cm), and high MBV (23.78 cm²), with death occurring at 10 months of follow-up.

Based on the presence of high MBV (≥ 19.36 cm³ for PFS and ≥ 48.42 cm³ for OS), the three risk categories significantly distinguished by the combination of high TMTV (≥ 69.54 cm³ for both PFS and OS) and high Dmax (≥ 27.56 cm for PFS and ≥ 31.23 cm for OS) were further stratified (**Figure 4**):Group 1 (no risk factors) was further stratified by MBV into a subgroup with favorable prognosis (n = 126, events = 0, 100% 5-year PFS; n = 154, events = 3, 98.1% 5-year OS) and a subgroup with poorer prognosis (n = 23, events = 3, 87.0% 5-year PFS; n = 2, events = 0, 100% 5-year OS). The two subgroups showed a significant difference in PFS (P < 0.0001), but no significant difference in OS (P = 0.843). In Group 3 (with both risk factors), patients with low MBV (n = 9, events = 4, 0% 5-year PFS; n = 27, events = 11, 38.6% 5-year OS) and high MBV (n = 65, events = 55, 10.1% 5-year PFS; n = 45, events = 39, 5.7% 5-year OS) showed significant differences in both PFS (P = 0.015) and OS (P < 0.0001), with an overall significance of P = 0.038. In Group 2 (with one risk factor), no significant differences were found in PFS (P = 0.767) or OS (P = 0.553) between patients with low MBV (n = 54, events = 12, 74.9% 5-year PFS; n = 65, events = 12, 75.6% 5-year OS) and those with high MBV (n = 34, events = 8, 72.6% 5-year PFS; n = 18, events = 2, 78.7% 5-year OS). **Figure 5** shows representative ^18^F-FDG PET maximum intensity projection (MIP) images of patients from Group 1 and Group 3 stratified by high/low MBV.

## 4 Discussion

Bulky disease has been proposed as a prognostic risk factor distinct from both the IPI and NCCN-IPI in the Cotswolds (25) and Lugano Classification (23). These two conferences defined and measured bulky disease by the maximum long diameter of the largest lesion. This study highlights the prognostic significance of a novel semiquantitative metric, MBV, measured by ^18^F-FDG PET/CT for characterizing bulky disease volume in newly diagnosed DLBCL patients in the R-CHOP era. Specifically, the following findings were noted (1): MBV was identified as an independent predictor of both PFS and OS in DLBCL patients prior to R-CHOP initiation, regardless of IPI, NCCN-IPI, and Dmax. Notably, MBV maintained independence from TMTV for PFS, though this independence was not observed for OS (2). High MBV correlates with poorer prognosis. The combination of MBV and Dmax enables risk stratification, with efficacy comparable to that of TMTV combined with Dmax (3). MBV reclassifies patients with high TMTV and high Dmax into a favorable-prognosis subgroup (no longer poor PFS/OS). For low TMTV and low Dmax, MBV identifies those with worse PFS (not OS). It fails to further stratify patients with only high TMTV or Dmax for PFS/OS.

At the initial Cotswolds Meeting, bulky disease was proposed as a poor prognostic indicator distinct from the IPI score, where a lymph node or lymph node mass ≥ 10 cm in maximum diameter was recorded as “bulky” (denoted by “X”) (25). Measurement modalities primarily included Computed Tomography (CT), Magnetic Resonance Imaging (MRI), and even chest X-ray. In the 2014 Lugano Classification, CT was recommended for measuring MTD of bulky disease, whereas chest X-ray was no longer recommended (23). Multiple studies have shown that in the rituximab era, the recommended MTD for measuring bulky disease in NHL varies (31, 32), no longer limited to the previously recommended 10 cm. Limited evidence suggests that for DLBCL in the rituximab era, the threshold is 6 to 10 cm (24). However, none of these recommended sizes have been validated in the current R-CHOP treatment era (23). The optimal cutoff values for MTD in this study were 4.95 for PFS and 5.15 for OS, which were lower than those in previous studies. This discrepancy might be attributed to differences in the study populations: approximately 66.1% (26) of the participants in the prior study had advanced-stage disease (III/IV), compared to 58.9% in our study.

^18^F-FDG PET/CT provides more information related to tumor metabolic activity compared with CT alone. For DLBCL patients, ^18^F-FDG PET/CT has been established as a staging procedure at diagnosis (33). Recent advances in ^18^F-FDG PET/CT imaging have shown that TMTV, as a surrogate for tumor cell burden, exhibits strong prognostic value in baseline DLBCL (10, 11, 13). Dmax, proposed as a dissemination feature in 2019, was found to complement TMTV in further stratifying baseline DLBCL patients (15). Subsequently, in multiple studies including those by our team, Dmax was identified as a strong prognostic predictor for DLBCL patients (14, 16), representing tumor dissemination. The conclusion that Dmax and TMTV, reflecting tumor dissemination and burden respectively, can complementarily stratify risk in baseline DLBCL was further validated in patients from the REMARC trial (NCT01122472) (14). The complementary prognostic value of Dmax and TMTV in DLBCL patients is consistent with previous findings. However, this study differs in that prior research was confined to high-risk patients with Ann Arbor stage III–IV disease (14), limiting applicability to all baseline DLBCL populations. By contrast, our study included patients with Ann Arbor stages I–IV, stratifying them into three groups based on the presence of high Dmax or high TMTV risk factors. Significant differences in both PFS and OS were observed among the three groups, validating the complementary role of Dmax and TMTV across all stages of baseline DLBCL and confirming their utility in newly diagnosed patients regardless of disease stage.

MBV, proposed as the metabolic tumor volume of the largest lesion, has been scarcely studied in DLBCL. In the limited existing research, it has been identified as an effective tool for stratifying baseline DLBCL patients (26, 27). Patients with high MBV had significantly worse PFS and OS than those with low MBV, consistent with the findings of this study. This may be attributed to the size effect of tumor drug absorption, where an increase in tumor size reduces drug concentration in tissue fluid (34). This could result in poorer drug absorption and worse prognosis in larger tumors. The study also demonstrated that MBV, rather than MTD, serves as a robust prognostic predictor for baseline DLBCL patients (26), consistent with the findings of this research. To mitigate the contingency associated with cutoff values, this study additionally employed the most widely recommended 10 cm (23, 25) as the MTD threshold for identical Cox proportional hazards regression analysis. Results showed that MBV retained significant prognostic value in both univariate and multivariate analyses, whereas MTD did not (**Supplementary Table 2**). This discrepancy may be attributed to the fact that MTD only measures the maximum diameter of the largest lesion, while MBV, as the metabolic tumor volume of the largest lesion, provides more metabolic activity information about bulky disease than MTD. This further indicates that MBV may be more suitable than MTD for characterizing bulky disease.

In the correlation analysis, this study found that MBV was very strongly correlated with TMTV and strongly correlated with MTD. We speculate that this may be because MBV represents the metabolic volume of the largest lymph node lesion, such that when a single lymph node is larger, MBV accounts for a greater proportion of TMTV, which may explain the very strong correlation between MBV and TMTV. Similarly, when MBV is larger, MTD tends to be larger, which explains the strong correlation between MBV and MTD.

Previous studies (26) have found a complementary relationship between MBV and TMTV, with MBV being independent of TMTV for OS, which differs from this study. This study found that when Dmax was added, MBV, Dmax, and TMTV were all independent predictors for PFS in DLBCL patients, while for OS, Dmax and MBV remained independent, but TMTV did not. This indicates that MBV is an independent prognostic predictor distinct from Dmax, and similar to the previous study, the presence of MBV may be complementary to TMTV.

Given that MBV was independent of Dmax in multivariate analysis, this study demonstrated that the combination of MBV and Dmax could also stratify baseline DLBCL patients into three groups, with significantly different prognoses among the groups. The combination of high Dmax and high MBV identified a larger number of patients with PFS events (N = 81), and the same number of patients with OS events (N = 64) as the TMTV-Dmax combination. To further investigate the relationship between bulky disease characteristics, lesion distribution, and total metabolic tumor burden, this study used MBV to further stratify the three groups defined by the two risk factors of Dmax and TMTV. The results showed that MBV had the ability to further stratify high-risk patients with both high TMTV and high Dmax and low-risk patients with neither risk factor. It can further subdivide patients originally considered high-risk (high TMTV and high Dmax) into subgroups with low-risk PFS and OS, and further subdivide low-risk patients (low TMTV and low Dmax) into subgroups with high-risk PFS. We speculate that this may be because TMTV represents the total tumor burden but cannot characterize the size and condition of individual lymph node lesions, while Dmax only reflects tumor distribution without indicating tumor burden. Therefore, when both TMTV and Dmax are low, the presence of a high MBV—likely due to poor drug absorption—results in relatively high PFS after treatment, though this does not apply to OS. This discrepancy might be attributed to the long-term effect of targeted intensive radiotherapy for relapsed bulky disease (35, 36)(Note: The number of patients receiving intensive radiotherapy in this study is to be confirmed.). Among patients with low TMTV and low Dmax, those with high MBV can still achieve favorable OS outcomes due to such interventions. When both TMTV and Dmax are high, patients with smaller MBV exhibit better PFS and OS outcomes. This suggests that when lymphomas are both small in volume and diffusely distributed (i.e., without bulky disease), patient prognosis is improved. This may be attributed to the fact that small, dispersed lymphomatous lesions allow for sufficient drug concentration in tissue fluid as chemotherapy agents circulate in the bloodstream (34), thereby leading to favorable PFS and OS outcomes. When only one of TMTV or Dmax is high, MBV fails to stratify patients for either PFS or OS. This may be attributed to the heterogeneity of DLBCL (37, 38), such that patients lacking either high TMTV or high Dmax are subject to a broader range of prognostic factors, and MBV does not serve as the sole or dominant prognostic factor in these populations.

The IPI is a major and classic clinical scoring system for lymphomas (6). Subsequently, in the R-CHOP treatment era, the NCCN-IPI was developed using the National Comprehensive Cancer Network (NCCN) database to enhance the IPI for newly diagnosed DLBCL patients receiving R-CHOP therapy (7). This study found that MBV is independent of both IPI and NCCN-IPI, an aspect not addressed in previous research. This indicates that in the R-CHOP treatment era, MBV not only characterizes bulky disease better than the historical MTD but also likely provides complementary and additive value to the IPI, NCCN-IPI, and their components.

This study has several limitations. First, this study is a retrospective single-center investigation. Given that retrospective analyses rely on existing clinical records and imaging data, they are inherently prone to potential selection bias and residual confounding factors. Thus, the value of MBV warrants further validation in large-sample, multi-center prospective studies. Second, since ^18^F-FDG PET/CT is not mandatory for baseline assessment, differences in patients’ economic status and physicians’ preferences inevitably introduce selection bias. Third, the pathological and biochemical mechanisms by which high MBV leads to poor prognosis in the context of R-CHOP first-line chemotherapy may need to be further explored in future studies. Finally, regarding future directions, recent studies have employed a multi-layered approach that integrates PET/CT radiomics(TMTV,TLG,Dmax), circulating tumor DNA (ctDNA) levels, and molecular clusters derived from liquid biopsy into a novel three-factor prognostic model. This model has been shown to stratify patient outcomes more accurately than clinical prognostic biomarkers (39). As another PET/CT parameter, whether MBV can further enhance the prognostic predictive value for patients with DLBCL when combined with ctDNA-based liquid biopsy warrants further investigation in future studies.

## 5 Conclusion

MBV measured by baseline ^18^F-FDG PET/CT, an indicator of metabolically active bulky disease, serves as an independent prognostic factor for patients with DLBCL. Combining MBV with Dmax improves risk stratification in patient staging. MBV, Dmax, and TMTV complement each other, highlighting that indicators of bulky disease should not be overlooked when considering total tumor burden and tumor dissemination.

## Data Availability

The raw data supporting the conclusions of this article will be made available by the authors, without undue reservation.
