# Metabolic Bulk Volume is an independent prognostic factor and facilitates identifying high risk cases for DLBCL patients treated with the R-CHOP

**DOI:** 10.3389/fonc.2026.1811308

**Published:** 2026-03-13

**Authors:** Silu Cui, Panpan Luan, Yuxiao Hu, Qi Jiang

**Affiliations:** 1 Jiangsu Province Hospital of Chinese Medicine, Affiliated Hospital of Nanjing University of Chinese Medicine, Nanjing, Jiangsu, China; 2 Department of PET/CT Center, Jiangsu Cancer Hospital and Jiangsu Institute of Cancer Research and The Affiliated Cancer Hospital of Nanjing Medical University, Nanjing, China

**Keywords:** 18F-FDG PET/CT, diffuse large B-cell lymphoma, metabolic bulk volume, prognosis, total metabolic tumour volume

There was a mistake in **Figure 4** as published. The label below “Panel B” of **Figure 4** was incorrectly marked as “PFS”. The correct label should be “time months”. The corrected **Figure 4** appears below.

**Figure 4:**
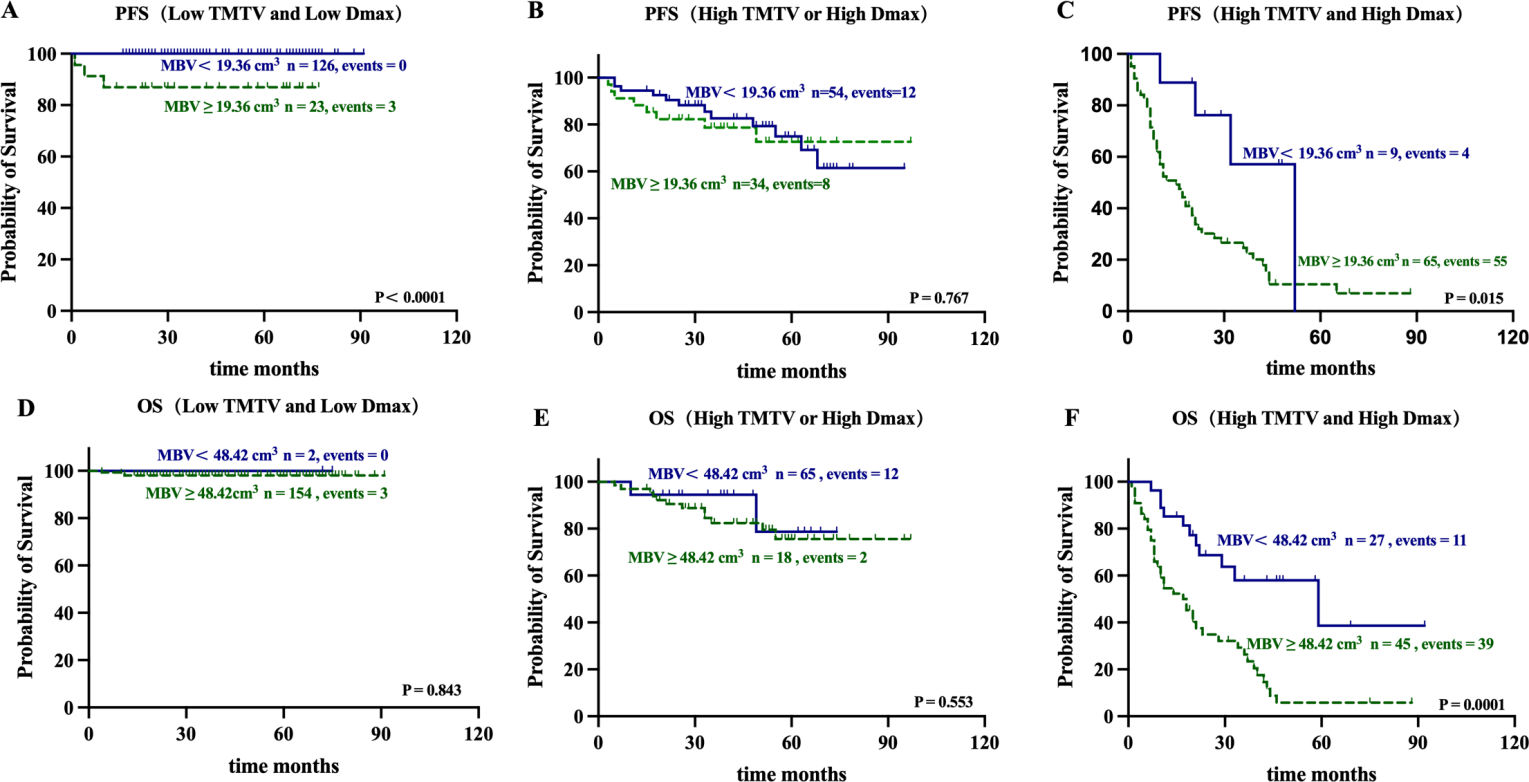
Kaplan–Meier estimates of PFS and OS for MBV (< 19.36cm3 vs. ≥ 19.36 cm3 and < 48.42 cm3 vs. ≥ 48.42 cm3, respectively) in TMTV-Dmax Combination Stratification (low TMTV and low Dmax: **(A, D)**; high TMTV or high Dmax: **(B, E)**; high TMTV and high Dmax: **(C, F)**.

There was a mistake in Figure 5 as published. “Panel A” of **Figure 5** contains two subfigures, and the first subfigure of “Panel A” was incorrectly duplicated from the first subfigure of “Panel B”. The corrected **Figure 5** appears below.

**Figure 5:**
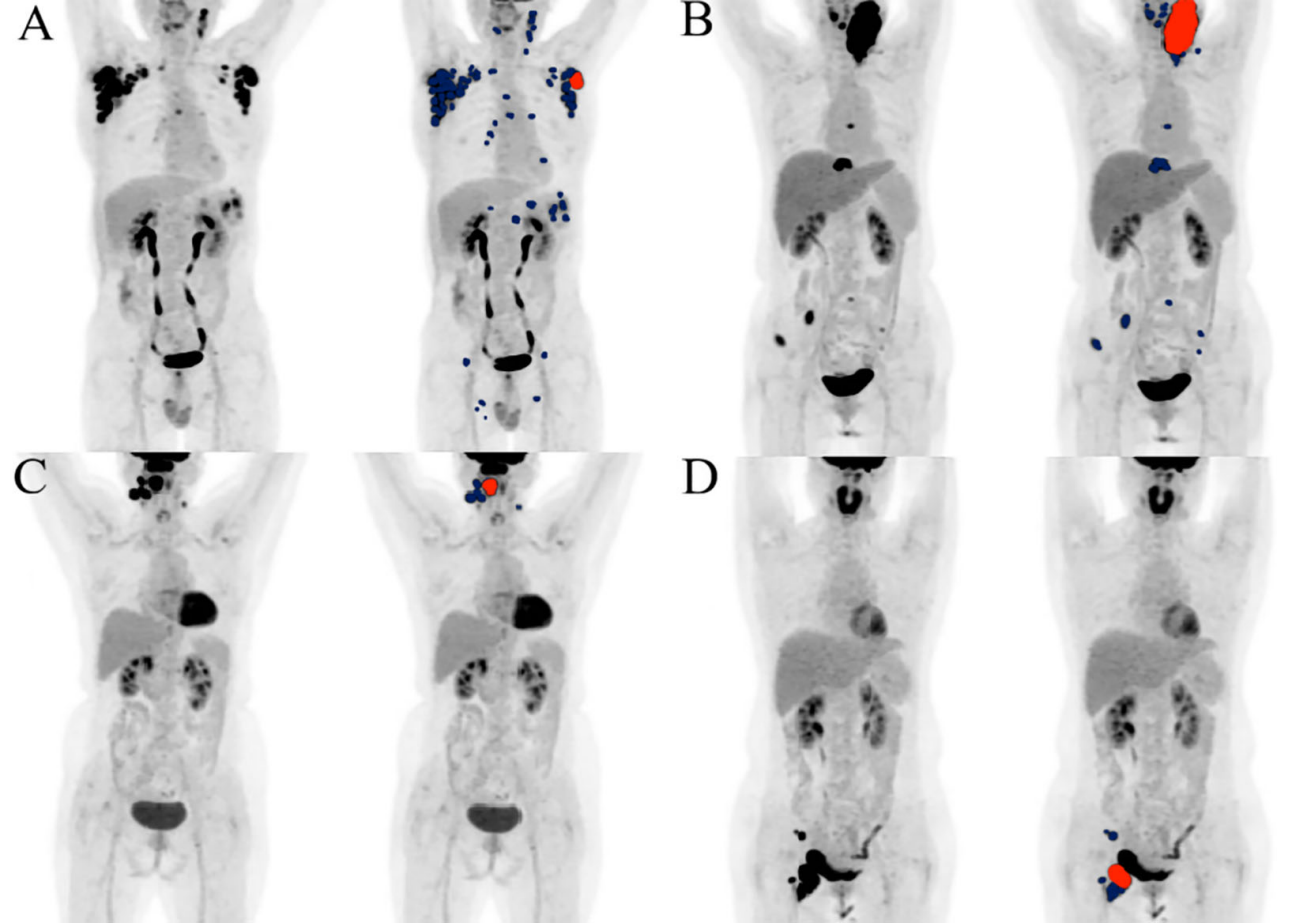
TMTV and Dmax from 18F-FDG PET/CT assess tumor burden and dissemination, respectively. MBV reclassified risk in two representative patients **(A, B)** with both high TMTV and high Dmax, as well as in another two (C, D) with both low TMTV and low Dmax. All involved lesions are indicated in blue, and the largest lesion used to derive MBV is highlighted in red. Patient **(A)** had high TMTV (99.71 cm^2^), high Dmax (68.8 cm), and low MBV (11.74 cm^2^), remaining progression-free and alive after 29 months of follow-up. Patient **(B)** showed high TMTV (100.23 cm^3^), high Dmax (59.24 cm), and high MBV (82.89 cm^2^); progression occurred at 3 months and death at 7 months of follow-up. Patient **(C)** had low TMTV (21.04 cm^3^), low Dmax (9.73 cm), and low MBV (6.30 cm^2^), remaining progression-free and alive after 83 months of follow-up. Patient **(D)** showed low TMTV (33.03 cm^3^), low Dmax (10.20 cm), and high MBV (23.78 cm^2^), with death occurring at 10 months of follow-up.

There was a mistake in the caption of **Table 4** as published. The title of **Table 4** was incorrectly stated as “Multivariate Cox regression analysis for OS and OS.” The correct title should be “Multivariate Cox regression analysis for PFS and OS”. The corrected caption of **Table 4** appears below.

“Multivariate Cox regression analysis for PFS and OS.”

The original version of this article has been updated.

