# Supplementary material for: Metabolic Bulk Volume is an independent prognostic factor and facilitates identifying high risk cases for DLBCL patients treated with the R-CHOP
Source: Front Oncol. 2026 Feb 3;16:1747186. doi: 10.3389/fonc.2026.1747186 (PMC12909205; doi:10.3389/fonc.2026.1747186)
Supplement: Supplementary file 1 [file DataSheet1.docx]

***Supplementary Material***

**1  Supplementary Figures and Tables**

- 1. **Supplementary Figure**


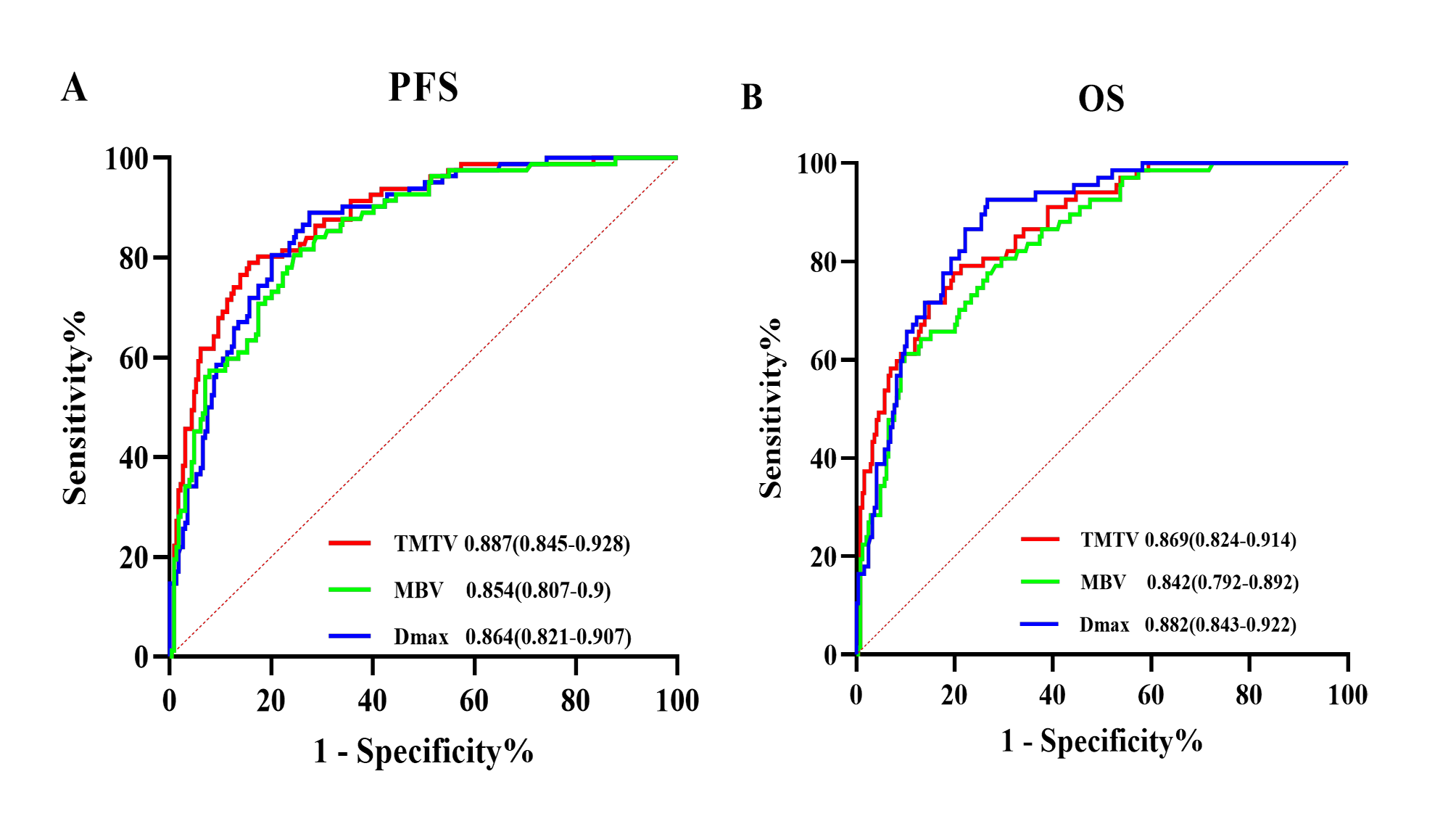
**Supplementary Figure 1** Receiver operating characteristic curves for PFS(A) and OS(B) for TMTV, MBV, Dmax.

**1.2 Supplementary Tables**

**Supplementary Table 1** Correlations between MBV and other metabolic parameters, Dmax, IPI, NCCN-IPI and components of the IPI

| Parameter | MBV | |
| --- | --- | --- |
|  | Pearson’s correlation coefficient (ρ) | P value |
| SUVmax | 0.076 | 0.180 |
| SUVmean | 0.110 | 0.053 |
| TMTV | 0.939 | ＜0.0001 |
| MTD | 0.707 | ＜0.0001 |
| Dmax | 0.166 | 0.003 |
| Age | 0.141 | 0.013 |
| Extranodal sites | 0.270 | ＜0.0001 |
| Ann Arbor Stage | 0.251 | ＜0.0001 |
| ECOG PS | 0.180 | 0.001 |
| LDH | 0.432 | ＜0.0001 |
| NCCN-IPI | 0.411 | ＜0.0001 |
| IPI | 0.333 | ＜0.0001 |

Pearson’s correlation coefficient (ρ) was classified as following: very weak (ρ= 0.0-0.19), weak (ρ= 0.20-0.39), moderate (ρ= 0.40-0.59), strong (ρ= 0.60-0.79), and very strong (ρ= 0.80-1.00).

**Supplementary Table 2** Multivariate Cox Regression Analysis for PFS and OS with MBV Cutoff at 10 cm³

| Variable | PFS |  |  |  | OS |  |  |
| --- | --- | --- | --- | --- | --- | --- | --- |
|  | HR | 95% CI | P value |  | HR | 95% CI | P value |
| Age | - | - | - |  | 1.243 | 0.715-2.163 | 0.441 |
| Ann Arbor Stage | 1.248 | 0.654-2.381 | 0.501 |  | 2.237 | 1.028-4.866 | **0.042** |
| LDH | 1.21 | 0.703-2.083 | 0.492 |  | 1.527 | 0.855-2.728 | 0.153 |
| EN | 1.621 | 0.967-2.716 | 0.067 |  | 1.78 | 1.009-3.142 | **0.047** |
| ECOG PS | 1.157 | 0.692-1.934 | 0.578 |  | 1.392 | 0.8-2.423 | 0.242 |
| TMTV | 3.318 | 1.513-7.277 | **0.003** |  | 1.296 | 0.549-3.057 | 0.554 |
| Dmax | 6.907 | 3.296-14.476 | **＜0.0001** |  | 11.723 | 4.481-30.669 | **＜0.0001** |
| SUVmax | 0.86 | 0.441-1.674 | 0.656 |  | 0.748 | 0.347-1.613 | 0.459 |
| SUVmean | 1.201 | 0.588-2.451 | 0.615 |  | 1.709 | 0.769-3.795 | 0.188 |
| MBV | 2.496 | 1.215-5.127 | **0.013** |  | 4.14 | 1.979-8.662 | **0.0002** |
| MTD | 0.894 | 0.514-1.556 | 0.693 |  | 1.243 | 0.715-2.163 | 0.088 |
